# Recent Advances in Understanding the Pathogenesis of Rheumatoid Arthritis: New Treatment Strategies

**DOI:** 10.3390/cells10113017

**Published:** 2021-11-04

**Authors:** Anna-Lena Mueller, Zahra Payandeh, Niloufar Mohammadkhani, Shaden M. H. Mubarak, Alireza Zakeri, Armina Alagheband Bahrami, Aranka Brockmueller, Mehdi Shakibaei

**Affiliations:** 1 Musculoskeletal Research Group and Tumor Biology, Chair of Vegetative Anatomy, Institute of Anatomy, Faculty of Medicine, Ludwig-Maximilian-University Munich, 80336 Munich, Germany; A.Mueller@med.uni-muenchen.de (A.-L.M.); aranka.brockmueller@med.uni-muenchen.de (A.B.); 2 Immunology Research Center, Tabriz University of Medical Sciences, Tabriz 5166-15731, Iran; Zpayandeh58@yahoo.com; 3 Department of Clinical Biochemistry, School of Medicine, Shahid Beheshti University of Medical Sciences, Tehran 1985717443, Iran; nsr.sbmu@gmail.com; 4 Children’s Medical Center, Network of Immunity in Infection, Malignancy and Autoimmunity (NIIMA), Universal Scientific Education and Research Network (USERN), Tehran 1419733151, Iran; 5 Department of Clinical Laboratory Science, Faculty of Pharmacy, University of Kufa, Najaf 1967365271, Iraq; shadenm.hasan@uokufa.edu.iq; 6 Department of Biology Sciences, Shahid Rajaee Teacher Training University, Tehran 1678815811, Iran; zakeri@yahoo.com; 7 Department of Biotechnology, School of Advanced Technologies in Medicine, Shahid Beheshti University of Medical Sciences, Tehran 1985717443, Iran; aarminaa@gmail.com

**Keywords:** rheumatoid arthritis, inflammation, auto-antibodies, autophagy, epigenetic, citrullination, biological agents, phytochemical, treatment

## Abstract

Rheumatoid arthritis (RA) is considered a chronic systemic, multi-factorial, inflammatory, and progressive autoimmune disease affecting many people worldwide. While patients show very individual courses of disease, with RA focusing on the musculoskeletal system, joints are often severely affected, leading to local inflammation, cartilage destruction, and bone erosion. To prevent joint damage and physical disability as one of many symptoms of RA, early diagnosis is critical. Auto-antibodies play a pivotal clinical role in patients with systemic RA. As biomarkers, they could help to make a more efficient diagnosis, prognosis, and treatment decision. Besides auto-antibodies, several other factors are involved in the progression of RA, such as epigenetic alterations, post-translational modifications, glycosylation, autophagy, and T-cells. Understanding the interplay between these factors would contribute to a deeper insight into the causes, mechanisms, progression, and treatment of the disease. In this review, the latest RA research findings are discussed to better understand the pathogenesis, and finally, treatment strategies for RA therapy are presented, including both conventional approaches and new methods that have been developed in recent years or are currently under investigation.

## 1. Introduction

Rheumatoid arthritis (RA) is a chronic inflammatory autoimmune condition with extensive degradation of cartilage and underlying bone that causes suffering in many people worldwide [1]. The joint damage could be prevented by early diagnosis and in turn could lead to improved long-lasting outcomes. A large body of data indicates that considerable permanent joint damage can occur within the first 2 years of disease onset; thus, optimal management of RA is essential during the first 3 to 6 months [2,3]. Therefore, reliable biomarkers are needed to provide early diagnosis, accurate prognosis, and improved management of disease. The pivotal role of immune cell infiltration into the joint followed by bone erosions are among the most significant characteristics of RA.

In relation to target antigens, numerous types of auto-antibodies have been classified as hallmarks of RA; two of them are the rheumatoid factor (RF) and anti-citrullinated protein antibodies (ACPA) [4]. Moreover, the contribution of genetic predisposition is thought to be about 50 to 60%, which has the most significant impact on the vulnerability to RA. The human leukocyte antigen (HLA) genes are the most forceful predisposing gene variants for developing RA, within the HLA class II histocompatibility antigen-DRB1-beta chain (HLA-DRB1) gene, a conserved amino acid sequence that is shared through multiple RA-associated risk alleles. Indeed, the HLA locus has been mainly associated with seropositive RA and with increased serum levels of antibodies (Abs) against citrullinated proteins [5,6].

The “shared epitope hypothesis”, as a possible basis for all diseases including HLA class II polymorphisms, which was proposed by Gregersen et al. in 1987, represents a key event and scientific advance in RA research. It says that certain alleles of the HLA-DR1 and HLA-DR4 molecules share a common amino acid sequence (the so called “shared epitope”), thus demonstrating a link between the pathogenesis of autoimmune diseases such as RA and these epitopes [7]. It should be noted that the pathogenesis of RA has a polygenic basis. Recent progress in genome-wide association studies (GWAS) has enhanced our knowledge of the genetic susceptibility underlying RA, introducing more than 100 genetic loci associated with an elevated risk for RA development [5,6].

Another missense risk variant in RA may be the R620W mutation in Protein tyrosine phosphatase, non-receptor type 22 (PTPN22) (derived from 1858C→T-cells) [8], encoding protein tyrosine phosphatase, an enzyme expressed in hematopoietic cells that negatively regulates antigen receptor signaling in B- and T-cells [9]. In addition, it has been reported that the R620W risk allele acts as a gain-of-function variant [10] and both T-cell receptor (TCR) and B-cell receptor (BCR) signaling is reduced in cells carrying this risk allele [11].

As mentioned earlier, the presence of auto-antibodies, such as RF and ACPA, is a characteristic feature of RA. They precede the commencement of disease manifestations and predict progression to the classic seropositive type of RA [2,12]; that is why these Abs are assumed to play a major role in RA’s pathogenesis. Since RA is a multifactorial disease, its development can not only depend on genetic conditions, but also on serological alterations as well as environmental factors. Great resources have been invested in understanding the potential influence of specific environmental factors, such as smoking, periodontitis, specific infections, lack of sunlight, or processed foods [13,14]. Furthermore, air pollution is also a highly topical issue, as a case-crossover study has recently shown that there is an association between strong air pollution, an increase in the inflammatory parameter C-reactive Protein (CRP), and the occurrence of relapses of RA [15]. In addition, metabolic changes are thought to play a role, as altered pro-inflammatory bacterial composition of the oral [16], salivary, dental, and intestinal microbiome [17] has been found in RA patients. Due to the changes in the metabolic system of RA patients known as lipid paradox, their cardiovascular factors need to be closely monitored [18]. In this review, we summarized recent discoveries in RA pathogenesis and research regarding auto-antibodies, epigenetic and post-translational modifications, glycosylation, autophagy, and T-cells. In addition, we focused on the impact of these issues regarding potential strategies for RA therapy in the selection of therapeutic concepts and clinical endpoints.

## 2. Auto-Antibodies Involved in RA Pathogenesis and Development

Auto-antibodies such as RF and ACPA are routinely determined as serum markers in rheumatoid arthritis patients. As a third of RA patients are seronegative, these markers cannot be regarded as specific, and the use of these parameters is controversial. However, it is worth pursuing further research of auto-antibodies, as now about a third of seronegative RA patients have been shown to form Abs associated with RA, including IgA isotypes of RF and ACPA, as well as RA33 Abs [4,19].

### 2.1. The Role of RF in Pathogenesis and Its Clinical Significance

B-cells aggregating in lymphoid follicles and germinal center-like structures of the inflamed RA synovium produce RF auto-antibodies that interact directly with the Fc region of accumulated IgG. The ranges of RF-based tests in RA patients are reported to be 60 to 90% regarding sensitivity and 48 to 92% for specificity, respectively [19]. Furthermore, evidence supports the pathogenic essence of RF and its involvement in the RA pathophysiology. Low-affinity IgM RF is produced by the introduction of immune complexes and polyclonal B-cell activators, such as lipopolysaccharides of bacteria and Epstein-Barr virus (EBV) [19]. In the pathogenic process of RA, high-affinity RF (in synovial fluid of joints) is involved in inflammation and antigen trapping. Interestingly, RF could locally induce immune complexes at synovial inflammatory sites, which could be followed by complement activation and leukocyte infiltration. In addition, RF acts as an important indicator in differential diagnosis and prognosis prediction for patients with arthritis. The RF isotypes may be present in the pre-clinical stage years before the onset of RA [19,20].

It has also been reported that antibody isotype production starts with IgM, followed by IgA, and finally IgG isotype [21]. It should be noted that a high RF titer is strongly associated with an unfavorable prognosis, more hostile articular disease, enhanced disease activity, and diminished remission rates, higher prevalence of extra-articular manifestations, and augmented morbidity and mortality. However, RA can be categorized as seropositive or seronegative depending on the presence or absence of RF [21,22].

In addition, the IgM RF can frequently be detected through various assays using citrullinated peptides, most commonly by the enzyme-linked immunosorbent assay (ELISA). Here, the most commonly used method is the CCP2 with undisclosed specificity. Advanced RF serology could be an important feature for clinical use to identify patients at an early stage of disease and to find subgroups of patients that are in need of active therapy [23].

### 2.2. The Role of Anti-Modified Protein Antibodies in RA

Besides RF, auto-antibodies against post-translationally modified proteins (anti-modified protein Abs or AMPAs) are also a hallmark of RA [24]. A wide range of AMPA classes binding to various modifications of proteins, including citrullination, carbamylation, and acetylation, have been characterized in RA [24,25]. In the following section, we focused on some of them.

#### 2.2.1. Anti-Citrullinated Protein Antibodies (ACPA)

Citrullination of proteins is a common physiological process in which a large number of proteins become citrullinated. However, citrullination of proteins can also occur in the process of inflammation, whereby the enzyme peptidyl arginine deiminase (PAD) interacts with specific binding sites on proteins and converts their exposed arginine side chains into citrulline [26]. While mammals have five PAD isozymes, only four of them are catalytically active (PAD 1, 2, 3, 4), whereby PAD2 and PAD4 are the most relevant considering RA, because they are overexpressed in immune cells [27,28]. The process of citrullination is calcium-dependent, because PADs are usually rather inactive until there are relatively high calcium concentrations [29]. However, as soon as PADs are activated, they start to citrullinate numerous proteins. Up to now, fibrinogen, α-enolase, filaggrin, vimentin, type II collagen (CII), and fibronectin are among the proteins that have been identified as targets of citrullination [30]. The citrullination process, converting a positively charged arginine to polar uncharged citrulline, can influence hydrogen bonding and ionic interplay, and therefore interferes with organized protein structure, possibly leading to destabilization or even suppression of inter- and intramolecular interactions [29,31,32]. In addition, Lundberg et al. showed that CII citrullination is a dominant mechanism for enhancing autoimmunity and indicated that the severity of arthritis correlates with the expression of PAD4 and the amount of citrullinated proteins at arthritic sites [33]. As a response to citrullinated proteins, B-cells induce the production of ACPA. This immunological response starts from fine specificity and epitope spread, to titer increase, isotype alteration, and immunological response maturation. The process continues with the elaboration of Abs structural diversity and consequently might lead to the activation of further immune effector procedures. The ACPA response relies on different isotypes, including IgG, IgA, and IgM [34], whereby elevated IgM and IgA levels are detected in approximately 60% of patients [35,36]. Moreover, the identification of ACPA plays a crucial role in understanding RA pathogenesis. The available data are consistent with the idea that there is a correlation between ACPA-positive and -negative RA and various genetic and environmental backgrounds, and this fact indicates that multiple pathophysiological processes might be involved in the disease’s subsets [36]. To note, RA patients display a non-standard humoral immune response against citrullinated proteins expressed at all inflammation sites. So, the citrullination process is implemented in a wide range of inflammatory tissues, and this property confirms that citrullination is an inflammation-associated process that should usually be tolerated by the immune system. It is known that in different physiological mechanisms such as the apoptosis signaling pathways, intracellular calcium concentration increases to levels far higher than physiological status. This increase ultimately terminates in PAD enzyme activation [37]. Moreover, auto-antibodies may be produced following the exposure of citrullinated peptides in the immune system of especially genetically predisposed individuals. In the long term, up-regulated pro-inflammatory cytokines form a complex immune response and drive chronic inflammation as a typical manifestation in RA. Thus, ACPA have been emphasized and according to their specificity, it has been shown that they can be divided into different classes [30,36].

On one hand, there is a class of “promiscuous” ACPA that is highly specific only to the citrulline side chain but can interact with a wide range of protein epitopes as long as there is no other surrounding side chain that could directly inhibit the interaction. On the other hand, there is a class termed “private” ACPA that interacts with the citrulline side chain as one of many side chains in the epitope, and these Abs are highly specific, even when binding in vivo [38]. In the private ACPA, citrulline recognition is similar to the proximal amino acid side chain identification, which facilitates protein-specific interaction. Notably, although promiscuous ACPA are more likely to predominate in sera (both before and after RA onset), no pathogenic bioactivity has been conclusively identified for them so far. However, private ACPA contribute to the specific recognition of citrullinated epitopes of joint proteins, supporting the notion that private ACPA are arthritogenic. In fact, ACPA tend to migrate into joints through association with their tissues and increase complex immune formations in situ, leading to bone erosion, pain, and arthritis [38,39].

Arnoux and co-workers demonstrated in a mouse model that ACPA production can be triggered by PAD immunization. Based on their results suggesting that a “hapten-carrier mechanism” is involved in ACPA synthesis, they were also able to lay the groundwork for a new potential mouse model of ACPA-positive RA [40]. More recently, together with Auger and coworkers, they were able to confirm their design by studying the peripheral blood of RA patients. They found that only patients with RA showed both an antibody and a T-cell response to the PAD4 enzyme, resulting in increased ACPA levels. Based on their findings, they eventually designed the “hapten-carrier model” of RA, in which PAD4 acts as a carrier, while the haptens are represented by the citrullinated proteins. Their findings not only further highlight the central role of PAD4 in immunological processes that ultimately lead to the autoimmune disease RA, but also contribute to better perspectives in RA prevention through PAD peptide tolerance [41].

#### 2.2.2. Anti-Carbamylated Protein Antibodies

Among RA patients, several auto-antibodies have been identified, including anti-carbamylated protein (anti-CarP) Abs, anti-PAD, and anti-malondialdehyde Abs. Among Abs targeting anti-CarP, extensive studies have been conducted [42,43]. Carbamylation is a non-enzymatic post-translational modification, whereby the isocyanic acid interacts with free amino groups of amino acids. Although anti-CarP Abs could be found prior to disease onset, they have been detected and analyzed in a significant number of patients with RA and other health conditions. However, it was reported in a study by Lo et al. that many RA patients did exhibit ACPA reactivity but did not represent an anti-CarP positive group in a majority of cases, underlining that anti-CarP auto-antibodies are not necessarily involved in the association with RA, and thus are not absolutely RA-specific [44]. It should also be noted that the anti-CarP auto-antibodies can even be present in RA patients that are seronegative for both ACPA and RF [45,46]. In addition, carbamylation is associated with decreased functionality of various enzymes and hormones. Furthermore, extensive carbamylation could stimulate an autoimmune feedback against carbamylated proteins in susceptible individuals, and it has been demonstrated that primary immune responses, chemotaxis, activation of T-cells, antibody production, and IL (Interleukin)-10-, IL-17-, and interferon (IFN)-γ-production could be induced by carbamylated proteins. Moreover, T-cell activation and extensive antibody response can facilitate the recognition of carbamylated and citrullinated peptides in the joints, which may end with erosive arthritis development [43]. It should be noted that carbamylated and citrullinated peptides complementarily contribute to elicitation of the autoimmune responses [47].

The immune-triggering impact of carbamylation increases the arthritogenic features of citrullinated peptides; this property represents a novel mechanism of action for the pathogenesis of autoimmune arthritis [43]. Recently, the existence of anti-CarP Abs was shown to correlate with elevated disease activity and significant disability in patients with RA over time [48]. To note, statistically significant correlations were revealed in ACPA-positive and -negative patients [49]. For instance, the presence of anti-CarP Abs in arthralgia patients suggests an ACPA-independent development of RA. Given these observations, it is not clearly recognized whether the anti-CarP Abs are rather a cross-reactive effect of ACPAs or whether they represent an independent priming event. On this background, anti-CarP Abs can be considered as helpful indicators in identifying potential RA-patients in some cases [50], but as studies have shown, their properties to act as a biomarker for diagnosing RA are limited [51]. Since the absence of anti-CarP Abs has been observed in patients with other inflammatory rheumatic diseases as well as in healthy individuals [52], its occurrence can be considered specific for RA to a limited extent, but as the results of a large study by Lo et al. recently showed, in which more than 4.6 million peptides were analyzed, there are more opportunities to identify unexplored ACPA epitopes that are further specific for RA [44]. Overall, it can be concluded that the combination of anti-CarP Abs, ACPA, and RF are particularly beneficial for early diagnoses of RA patients [53].

#### 2.2.3. Anti-PAD4 Antibodies

The citrullinating enzyme peptidylarginine deiminase 4 (PADI4) was identified as a susceptibility gene in a case–control study by Suzuki et al., using single nucleotide polymorphisms (SNPs) and also examining serum from individuals suffering from RA [26]. Moreover, Suzuki and coworkers showed in their study that the described susceptibility gene is associated with higher levels of citrullinated peptide Abs in RA patients [26]. Interestingly, this change can already be detected in the early stages of RA in humans, whereupon the immune system collapses and severe autoimmunity problems occur [26]. Recently, it has been revealed that the auto-citrullination of PAD4 as a control mechanism might inactivate the enzyme by changing the enzyme’s structure [54], leading to an increase in its recognition by human auto-antibodies [37]. Furthermore, anti-PAD4 auto-antibodies can be found in about 35% of RA patients (with more than 95% specificity). Anti-PAD4- Abs have predictive and prognostic value for RA patients because they can target and activate PAD, enhancing the enzyme’s catalytic efficiency by reducing its calcium requirement. The functional effects of anti-PAD4 Abs depend on their interaction with various PAD4 substrates or the epitopes they bind. Moreover, it is suggested that anti-PAD4 Abs may have a negative impact on the activity of PAD4 by disrupting its dimerization, which is essential for the full activity of the enzyme and could potentially lead to joint degradation by human proteases such as MMPs. Disruption of PAD4 may also enhance the inflammatory response by supporting activation of the complement system and triggering secretion of cytokines via Fc receptors on immune cells [55]. However, anti-PAD4 Abs had no significant association with the ACPA levels and the disease activity in patients. However, since they are commonly identified following the ACPA appearance, they are suggested to be associated with the existence of ACPA [56].

#### 2.2.4. Anti-b-Raf and Anti-RA33 Antibodies

Anti-b-Raf auto-antibodies activate b-Raf kinase function, which could lead to the production of pro-inflammatory cytokines and joint inflammation. These Abs are produced by approximately 21–32% of RA patients [2]. Interestingly, almost one third of anti-CCP2-negative RA patients are anti-b-Raf-(serine/threonine-protein kinase b-Raf) positive [2,57]. So, these are one of the most interesting auto-antibodies for classifying ACPA-negative RA patients [58]. Moreover, Abs to the heterogeneous nuclear ribonucleoprotein A2/B1 (Anti-RA33) and T-cells targeting RA33 can also be associated with the autoimmunity and inflammation [59]. They can exert their effects via formation of immune complexes or through induction of cytokine secretion that might trigger and develop the pathogenic process. The pathogenic functions of anti-RA33 Abs are yet to be elucidated; they could be detected in the earliest stages of RA or even years before the onset of the clinical manifestations. Indeed, there is also evidence that these Abs do not contribute significantly to bone erosions or disease activity [60].

## 3. The Role of Epigenetic Modifications and Glycosylation in RA

Epigenetics is the study of cellular and physiologically reversible changes in gene function while the DNA sequence does not change. In other words, epigenetics involves inherited changes in gene expression without modification of genetic structures. Epigenetic mechanisms are sensitive to external stimuli, and epigenetic alterations are crucial for the development of immune cells and the modulation of their differentiation processes. These processes are highly pivotal in antibody maturation and the auto-antibody response [58,61]. Once selected for proliferation and survival, B-cells differentiate into either plasma or memory cells depending on different stimuli to which they are exposed. For example, memory B-cells differentiate into plasma cells in response to stimulation by antigens and Toll-like receptors (TLRs) among other factors [62,63]. Epigenetic mechanisms include gene expression regulation through DNA methylation, post-translational histone modifications, and non-coding RNAs (ncRNAs). Unlike genetic mutations, epigenetic changes are reversible, making them a suitable therapeutic target. Recently, the use of drugs (for example azacitidine, etinostat, vorinostat, tazemetostat, molibresib) that modify epigenetic changes has been reported in the treatment of several cancers, neurological conditions, and heart diseases [64,65]. As epigenetic modifications are essential in regulating gene expression, epigenetic-based therapies could be an important lever for RA diagnosis and management [62,63,66].

### 3.1. DNA Methylation and Demethylation

Methylation and demethylation regulate the expression of specific, tissue-dependent genes. It has been demonstrated that during the development of bone marrow B-cells and peripheral differentiation, progressive demethylation occurs in the B-cell genome [66]. In gene promoter and enhancer regions, DNA methyltransferases methylate cytidine to 5-methylcytosine and silence gene expression. Passive or active demethylation could de-methylate the 5-methylcytosine to its un-methylated form [66,67]. DNA demethylation arises during differentiation of B-cells to plasma cells, with DNA hypomethylation predominating in Prdm1 [68]. Moreover, the DNA methylation pattern is also changed in RA, leading to disease progression [69]. To note, the RA Fibroblast-Like Synoviocytes (FLS) and Peripheral Blood Mononuclear Cells (PBMCs) genes are hypo-methylated in RA patients [70]. Hypo-methylated loci have also been found in other RA-related genes such as the signal transducer and activator of transcription 3 (STAT3) [71]. Activation of this gene is associated with increased expression of IL-6, which plays a key role in the pathogenesis of RA [72]. Recent studies revealed a new methylation signature in T- and B-cells in early RA patients [66,71]. Alteration in the DNA methylation pattern in the early stages of RA development affected the disease progression. Moreover, the methylation level of the IL-6 promoter in PBMCs in RA patients is significantly lower than in healthy control patients [73].

### 3.2. Histone Modifications

Another important epigenetic change is the altered pattern of histone modification, which may play a role in the development of RA [74]. Furthermore, phosphorylation, acetylation, ubiquitylation, and sumoylation are different covalent post-translational modifications of histones. The balance between the histone acetylases and histone deacetylases is essential, but in RA patients, histone hyper-acetylation was observed [75]. Here, the histone H3 of FLS in RA patients was highly acetylated in the promoter region of IL-6 gene, and histone acetyltransferase (HAT) inhibitors, such as curcumin, have been shown to reduce the IL-6 secretion, indicating the importance of epigenetic mechanisms in RA pathogenesis [76].

Reversible acetylation is one of the most important modifications in lysine residues. This modification is regulated by the histone deacetylases (HDACs), which are involved in chromatin condensation, repression of transcription, T-cell subset differentiation, and T-cell-mediated autoimmune diseases [63]. Since the expression of a large number of genes is controlled by HDACs and they modulate a variety of protein functions through non-histone deacetylation, they may play a role in disorders and diseases. Indeed, dysregulations of HDACs in the form of abnormal levels leading to either increased or decreased activity have been found in several cells such as macrophages, FLS, and PBMCs in the context of RA [77]. As they directly correlate with higher disease activity, HDACs can be targeted as another treatment strategy [75,78]. Indeed, the use of HDAC inhibitors has been shown to be effective in the treatment of inflammatory diseases [79], and deletion of HDAC1 in T-cells even lead to complete protection of collagen-induced arthritis (CIA) in mice [80], which is a new treatment target in RA [81].

### 3.3. Glycosylation

Glycosylation is a posttranslational modification with significant effects on biological functions, and the modification may lead to an inflammatory response in the humoral immune system [82]. To note, it has been reported that patients with auto-immune diseases such as RA undergo different glycosylation patterns of total IgG [83]. Furthermore, fucosylation, galactosylation, and sialylation are different types of antibody glycosylation [84]. Several studies have shown the significance of glycosylation in the variable Fab regions of Abs [85], which occurs in more than 90% of IgG-ACPA [86]. However, the degree of glycosylation of the variable domains is much higher, and based on the type and composition of the glycans [87], antibody glycosylation could function in a pro- or anti-inflammatory way [88]. To note, the fucosylation level is increased in RA patients, especially during the chronic inflammation [89], but the sialylation level is reduced in IgG of these patients and also in mouse models [87,89]. Galactosylation and sialylation are both key players in the regulation of the antibody effector function [87,90]. The sialylation of Abs changes their structural conformation, reduces their affinity to FcγR, and consequently, their inflammatory effector functions. In ACPA, Fab domains are highly galactosylated and sialyated, and in RA patients, the pattern of Fc glycosylation changes in ACPA [87,89,91]. The addition of diverse N-glycan and glycosylation sites [86] on the variable domains of IgGs is correlated with the risk of developing RA transformations [92].

Recently, Zhipeng Su et al. employed linear ion-trap electrospray ionization mass spectrometry (LTQ-ESI-MS) to identify permethylated N-glycans IgG in RA patients. Their study showed that total purified IgG from RA has considerably lower galactosylation, lower sialylation, and higher fucosylation compared to that in healthy controls. Hence, in RA patients there should be a positive association between aberrant N-glycosylation and RF levels [93]. Recently, analysis of crystal structures of ACPA demonstrated that the V-domain glycans are positioned in the vicinity of the binding-pocket. Their dynamic modeling has shown the potential of V-domain glycans to interact with the antigen-binding regions. It is noteworthy that human Ramos B-cells carrying V-domain glycosylated B-cell receptors (compared to their non-glycosylated counterparts) undergo increased signaling after stimulation [93]. Furthermore, glycan-based nanoparticles recently have been used to boost the immune system [94].

## 4. Auto-Antibody Cross-Talk and T-Cells in RA

The RA pathogenesis typically occurs in synovial joints, where immune cells invade the synovium. In addition, the number of FLS in the underlying layer of the synovium significantly increases, leading to pannus formation. FLS observed in RA develop an aggressive phenotype that contributes to increased invasiveness in the extracellular matrix, leading to further joint damage. Moreover, RA FLS contribute to cartilage destruction and joint degradation by generating cytokines (IL-6, IL-8) and matrix degrading proteases (MMPs) that help maintain RA inflammatory disease. Due to the abundance of CD4^+^ memory T-cells in damaged joints of RA patients and the expansion of CD4^+^ clones in the synovial tissue of early disease, T-cell proliferation could be induced by local antigens. Moreover, the efficiency of co-stimulation blockade for CD80/CD86-CD28 interaction, such as cytotoxic T-lymphocyte-associated protein (CTLA)-4-Ig, highlights the importance of T-cells in RA pathogenesis [95]. The autoimmune disease of RA is generally associated with the major histocompatibility complex class II (MHC II) gene, particularly the DR alleles. These genes are the most essential in the so-called seropositive or classical RA. The identity or function of the MHC II has not been conclusively established in the disease. However, this association provides strong evidence that autoreactive T-cells are involved in early stages of RA pathogenesis. To note, B-cells are not required to activate T-cells, although T-cells are generally required to generate class-switched IgG-producing B-lineage cells [96,97].

Stastny et al. were the first to establish an association between RA and HLA-DRB1, and their report was further confirmed by GWAS [98,99]. This association ultimately led to the aforementioned “shared epitope hypothesis” as promoted by Gregersen and co-workers, a milestone in the further study of RA. According to the concept of this hypothesis, a five-amino acid sequence found in specific HLA-DRB1 alleles (shared epitope) is associated with increased RA susceptibility, contributing to the risk of RA disease [7]. On this basis, Hill and co-workers proposed for the first time that DRB1 alleles with the common epitope can elicit an autoimmune response to citrullinated RA antigens due to a significant increase in MHC-peptide interaction accompanied by an activated CD4^+^ T-cell response in HLA-DRB*0401 transgenic mice [100]. Later, a new citrullinated-CII peptide was discovered based on its ability to activate CD4^+^ T-cells from HLA-DRB1*10:01 positive individuals, resulting in the production of pro-inflammatory cytokines (IFN-γ, TNF, IL-17, IL-13, IL-10) [101]. In a large study by Sidney and co-workers, more than 200 citrullinated peptides of collagen II and vimentin were analyzed to investigate their binding properties. Here, 117 peptides were found to bind with significant affinity to the HLA-DRB1*01:01/HLA-DRB1*04:01 RA-associated shared epitope alleles. Against this background, it is now suggested that citrullination has an even stronger influence on T-cell recognition than HLA binding itself [102].

Besides the shared epitope, genes encoding the PTPN22, CD28, and CTLA4 (responsible for T-cell activation and differentiation) have also been shown to be associated with RA [5,103,104]. However, searching for immuno-dominant T-cell epitopes remains a major starting point in the field of RA study. The important subsets of CD4^+^ T-cell involved in RA include the T-helper cells (Th)1, Th17, regulatory T-cells (Treg), Tfh, and cytotoxic cells. In fact, in the synovial joint, macrophage activation is induced by Th1 cells and is characterized by an elevated capability for pro-inflammatory cytokine production including tumor necrosis factor (TNF) [105,106].

The abundance of Th17 cells in the peripheral blood of RA patients has been reported to vary from increasing to maintaining the status quo. In addition, according to the results of ex vivo peptide-HLADR-tetramer analysis, Th17-inducing cytokines are found in the synovial joint [107] and synovial IL-17 has been shown to induce bone resorption in RA patients [108]. Furthermore, Coutant et al. have shown that dysfunctional dendritic cells are not only associated with autoimmune diseases, but also contribute to the boosting of Th17 abundance through altered cytokine secretion. Briefly, cytokines are released that cause inadequate presentation of autoantigens to T-cells, resulting in a negative imbalance between T-helper cell types such as Th17 and Tregs [109], supporting the idea that Th17 cells are of great relevance in the development of RA. It has been recently suggested that IL-17-producing T-cells may play a role in the early stage of the disease or represent a major part in a subtype of RA patients [110]. Furthermore, in both, synovial fluid and tissues of RA patients, FOXP3^+^ CD25^+^ CD4^+^ Tregs are accumulated [111]. Moreover, ex vivo studies have confirmed the existence of CD4^+^ T-cells with cytotoxic potential (CD4^+^ CTLs) in various human viral diseases such as cytomegalovirus (CMV), EBV, influenza, human immunodeficiency virus (HIV-1), dengue, Hantavirus, and parvovirus B19 [112,113,114], although the amount of peripheral CD4^+^ CTLs is usually very low in healthy individuals, and CD4^+^ CD28 null T-cells (subset of proinflammatory T-cells) are not predominantly present in synovial fluid [115]. However, it has also been found that CD4^+^CD28^-^ T-cells are significantly increased in autoimmune diseases such as RA. Moreover, reports have shown that such a significant accumulation of the CD4+CD28^-^ T-cell subset is only found in CMV-positive individuals, in whom the CD4^+^CD28^-^ T-cell concentration was 22-fold higher compared with CMV-negative RA patients. This observation contributes to a possible link between CMV infections and the production of CD4^+^ CTLs in RA patients [116]. Repeated stimulation with antigens has been shown to be a classic feature of chronic inflammation and a compelling feature of CD4^+^ CTL formation [112].

Self- or cross-reactive T-cells can provide the necessary co-stimulation of B-cells to induce auto-antibody production. This kind of B-cell support appears to be the primary role of T-cells in various animal models of RA tested [117], since T-cells are not required for the disease perpetuation [118]. However, it has been shown that the activation of autoreactive T-cells is among the important factors that drive the autoimmune disease to a chronic stage [119].

**Figure 1:**
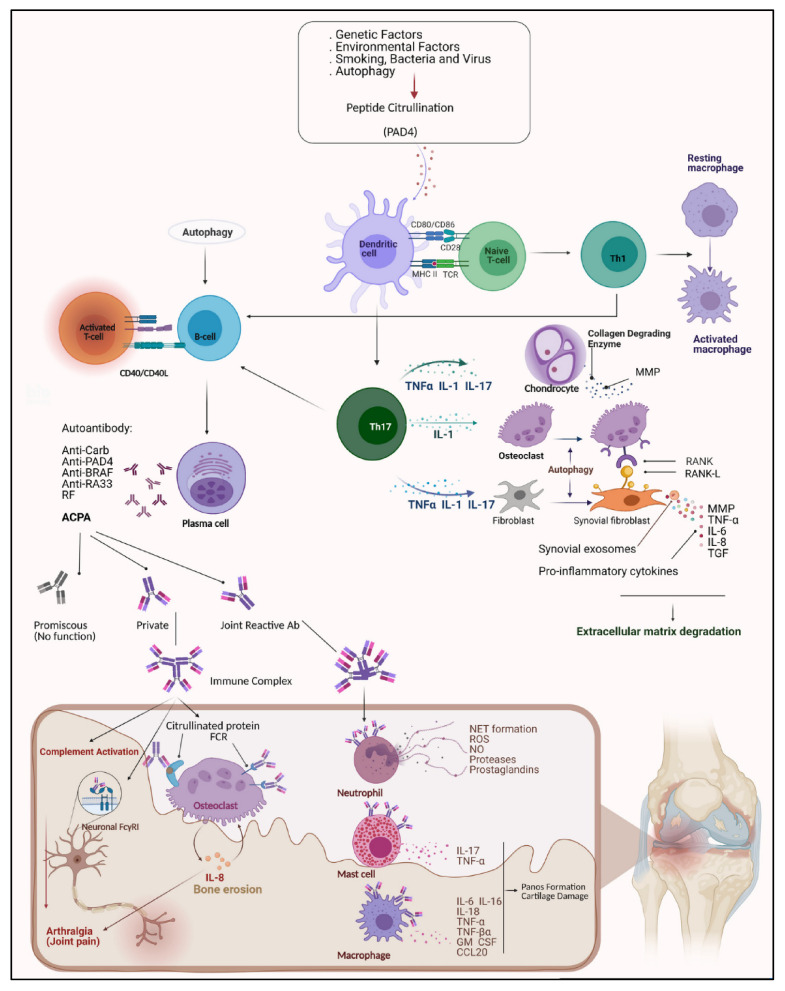
The etiology of rheumatoid arthritis. Multiple factors such as genetic background, smoking, bacterial infections, viral infections, and autophagy are involved in catalyzing the process of converting arginine to citrulline by PADI4 enzyme. Antigen presentation by the antigen presenting cells (APC) activates the naive T-cell, Th1, Th17, and Th2 cells. Th1 cells cause macrophage activation in the synovial joint by an elevated capability to secrete pro-inflammatory TNF. Th17 produces IL-17, IL1, and TNF-α, which effect chondrocytes, osteoclasts, and fibroblasts. Chondrocytes release collagen-releasing enzymes and Matrix-Metalloproteinase (MMP). Fibroblasts transform into FLS, which produce pro-inflammatory cytokines leading to destruction of the extracellular matrix. T-cells activate B-cells and plasma cells that secrete a variety of auto-antibodies. Auto-antibodies can attach to neutrophils and macrophages and lead to pannus formation and cartilage damage. They also can form an immune complex leading to joint pain and bone destruction.

It has already been demonstrated that TCRs have a great role in arthritis expansion. Therefore, some studies have focused on the TCR mutations that could lead to altered TCR signaling, increased numbers of autoreactive T-cells, and ultimately arthritis development [120]. In addition, endophilin A2 (EA2) has been identified as a regulator of TCR internalization, signal transduction, and down-stream T-cell effector functions, and the deficiency of this gene profoundly regulates arthritis and could lead to protection against autoimmunity [121], restricting the induction of autoreactive T-cells. Understanding its effects on the mechanism of T-cell activation could lead to new insights and therapeutic solutions for RA and all other T-cell-dependent inflammatory diseases [121]. The etiology of RA is presented in Figure 1.

## 5. The Role of Autophagy and Oxidative Stress in RA

Autophagy is a vital physiological process that directs cells to respond to damage by destroying useless and unessential self-components. Despite its role in human pathology, autophagy is involved in organelle turnover and elimination of protein aggregates [122]. During cellular stress, it is responsible for the degradation of intracellular components to produce adenosine triphosphate (ATP) and maintain essential cellular functions [123]. However, the process of autophagy can be a double-edged sword: To date, there is increasing evidence that autophagy can trigger autoimmune diseases [124], and by observing elevated levels in the synovial tissue of RA patients, it has been shown that autophagy promotes the development of RA [124,125]. The mTOR complex 1 (mTORC1), which acts as cellular energy level sensor, plays an important role in autophagy induction. It is inhibited by nutritional starvation, leading to activation of autophagy. Indeed, abnormalities in the PI3K/AKT/mTOR signaling axis have been found in RA patients [126], therefore, suppression of mTOR signaling could be an alternative approach for RA treatment [127].

Furthermore, Nedjic et al. have reported a high level of autophagy in Protein-tyrosine-kinases (PTKs), and this observation may suggest a role for autophagy in the development of the lymphocyte repertoire during thymic selection. Consistent with this study, selection of MHC II-restricted TCRs was recently shown to be altered in mice transplanted with ATG5−/− thymus. In addition, autophagy disturbance and lack of self-tolerance might be the cause of numerous autoimmune symptoms reported in these animals [128]. Autophagy could participate in a prolonged autoimmune response that promotes the survival of autoreactive and inflammatory cells, cytokine secretion, and citrullinated-antigen presentation [97]. In light of this, autophagy modulators could be considered as another therapeutic option to increase the efficacy of RA treatment [127].

### 5.1. Autophagy and Apoptosis

Based on the role of Th1 cells in RA, which when activated lead to osteoclast-regulated bone destruction, recent evidence suggests that autophagy may be involved in osteoclastogenesis. In particular, hypoxia, which triggers the activation of autophagy, appears to provoke osteoclast maturation [129]. In addition, it has been shown that Receptor Activator of NF-κB Ligand (RANKL) therapy could lead to up-regulation of markers related to autophagy. The knockdown of the cargo protein, p62, could decrease the expression of osteoclastogenesis-associated genes [130]. Indeed, the inhibition of autophagy has alleviated bone erosion and the number of osteoclasts in experimental arthritis mouse models [131]. These observations suggest that autophagy plays a crucial role in bone tissue breakdown. As a result, drugs targeting down-regulation of autophagy could be utilized in RA patients in order to prevent bone resorption [132].

Autophagy is a genetically controlled process that promotes cell survival under nutrient-deficient conditions, and the correlation between autophagy and apoptosis as two physiological processes alters the cell fate [133]. Since autophagy eliminates the damaged mitochondria, it plays a role in the reduction of reactive oxygen species (ROS) and defective DNA, accordingly hindering apoptosis [133,134]. It has been reported that caspase-dependent cleavage of Beclin-1 and its mitochondrial localization stimulate the secretion of pro-apoptotic factors from these organelles [135]. The balance between cell survival and death plays a key role in the pathogenesis and development of RA. Moreover, apoptosis helps to terminate the inflammation via blocking extreme immune cell activation and cytokine secretion. In fact, it has been demonstrated that apoptosis is down-regulated in RA tissue [136,137]. RA FLS undergo a multifaceted pattern of molecular changes leading to activation of RA-related genes and pathways (e.g., AP-1, NF-kB pathway) that are thought to be responsible for their aggressive and invasive phenotype [138]. The resistance of FLS to apoptotic induction may be responsible for the progressive deterioration of bone and cartilage tissues, and various intracellular processes such as autophagy may be involved. However, the interplay between autophagy and apoptosis still remains controversial [139].

More interestingly, it has been reported that induction of autophagy could lead to self-protection of RA cells from apoptosis and an increased lifespan [124]. In line with this hypothesis, endoplasmic reticulum (ER) stress could cause higher autophagy in RA patients compared to osteoarthritis (OA) patients [140]. Moreover, RA-FLS appear to be more resistant to cell death induction [141,142]. In fact, in RA patients’ synovial tissues, there was an inverse association between autophagy and apoptosis [141]. This property could confirm the association of autophagy with the apoptosis-resistant phenotype of RA synoviocytes. According to a recent report in the CIA mouse model, inhibition of autophagy could reduce synovial inflammation and increase synovial cell apoptosis by regulating the PI3K/AKT signaling pathways. Indeed, these data suggest that autophagy plays an essential protective role against apoptosis. Therefore, therapeutic approaches based on suppression of autophagy may have a significant impact on RA treatment [125].

### 5.2. Autophagy in Lymphocyte Homeostasis

Autophagy inhibition has been shown to impair T-cell activation [143], and deletion of anti-thymocyte globulin 7 (ATG7) decreases IL-2 mRNA levels and ATP production. This suggests that autophagy is necessary to guarantee the proper energy supply for T-cell activation. Related data have also been reported for B-lymphocytes [144]. The obtained results indicate that autophagy is important for B-cell maturation and maintenance of these lymphocytes’ reservoir in the periphery. In addition, RA as a systemic autoimmune disease is defined by the secretion of pathogenic auto-antibodies from plasma cells (PCs). Increased levels of these Abs could be associated with autophagy defects, and autophagy appears to be involved in the “PC differentiation program”, due to its activation during this process [145]. As a result, suppressing autophagy makes PCs more sensitive to cell death, halting antibody secretion. In consistence with these data, Conway et al. have achieved similar results, demonstrating the special role of autophagy in the PCs’ homeostasis [146].

Van Loosdregt has reported that the CD4^+^ T-cell population of RA patients treated with hydroxy chloroquine (HCQ) (an autophagy inhibitor) increased microtubule-associated proteins 1A/1B light chain 3B (LC3-II) levels and the number of autophagosomes compared to cells from healthy donors [147]. Hyperactivation of autophagy was found in CD4^+^ T-cells at sites of inflammation in the CIA mouse model. Moreover, arthritis symptoms were reduced in animal models injected with HCQ. Given these circumstances, it could be concluded that autophagy might preserve the autoreactive T- and B-cell populations to induce a sustained chronic inflammatory response in RA. However, more experimental evidence is required to confirm this hypothesis [147].

### 5.3. Autophagy and Citrullination

Autophagy appears to play a role in citrullinated peptide presentation and production of anti-CCP Abs in RA. In fact, Ireland et al. have shown that the antigen-presenting cells require autophagy for the successful presentation of citrullinated proteins, and the inhibition of autophagy stops this antigen presentation [148], whereas autophagy is not necessary to present unmodified antigens. Moreover, the expression of enzymes such as PAD and pro-autophagic factors such as nutrient disregard lead to an increased presence of citrullinated peptides in B-cells [149]. In a recent study, the expression of citrullinated proteins such as vimentin and α-enolase were promoted in FLS of RA patients following rapamycin treatment as an autophagy inducer. Furthermore, a direct correlation was found between LC3-II levels and anti-CCP titers in monocytes from early active RA patients [149]. This evidence highlights the role of autophagy activation in the breakdown of self-tolerance by sustained production of citrullinated peptides. In light of these facts, citrullination could be considered as a “biochemical marker of autophagy” [150].

### 5.4. Oxidative Stress

An imbalance in the regulation of oxidative stress has an essential role in the therapy of inflammatory autoimmune diseases via modulation of cell fate mechanisms. Furthermore, mitochondria are the major metabolic source of ROS. However, inflammatory stimuli could also activate the NADPH oxidase 2 (NOX2) complex to be an overriding source of ROS in specific cells [151]. To note, mitophagy (autophagy-mediated mitochondria degradation) maintains the proper balance of oxidative species. Nevertheless, the interplay between autophagy and oxidative stress is more complicated in the context of RA, and total oxidative status is higher in RA patients [152]. Moreover, the ROS level of neutrophils is positively correlated with disease activity in RA patients. Since ROS have been demonstrated to be involved in the formation of autophagosomes, ROS-mediated induction of autophagy might be pivotal in the resistance of synovial and peripheral RA T-cells against apoptosis and the production of citrullinated peptides in APCs [153].

In addition, neutrophil cytosolic factor 1 (NCF1, rs201802880, major/minor alleles: A/G, amino acid substitution: C to T from arginine to histidine) of the NOX2 complex (also known as p47PHOX), which is essential for ROS initiation, has been shown to play an important role in autoimmune diseases. In the human genome, the genomic structure of NCF1 is very complex, which is why it was excluded from GWAS. Furthermore, two non-functional pseudogenes with an almost identical sequence are located near the NCF1 gene, which shows varying copy numbers [154]. It most likely performs its functions by regulating peroxide levels. In addition, superoxide could be converted to peroxide or peroxynitrite anions when it reacts with nitric oxide (NO) in an aqueous environment [154]. Indeed, in cellular and immune responses, superoxide and peroxynitrites play a dual role. Olofsson and Hultquist et al. have previously shown that a single nucleotide polymorphism in NCF1 could result in a loss-of-function amino acid substitution [155,156] and that this loss of function may lead to an increased risk of developing arthritis. It has been shown that in mice, a superoxide defect caused by mutations in the NCF1 gene was found to cause arthritis and lupus [157]. A growing body of evidence suggests that an SNP in the NCF1 gene is linked with a range of different inflammatory and autoimmune conditions, each with different downstream pathogenic pathways regulated by NCF1 [158].

The task of identifying genes that underpin the quantitative trait loci (QTL) for complex diseases such as RA is difficult and time-consuming. Olofsson et al. discovered that a naturally occurring polymorphism of NCF1 controls the severity of arthritis by performing positional cloning of the Pia4 QTL in rats [155]. The disease-associated NCF1 allele reduces oxidative burst response and encourages arthritogenic T-cell activation. Treatments that stimulate the NADPH oxidase complex have been shown to promote arthritis development. Consequently, NCF1 is linked to a novel autoimmune mechanism that could lead to extreme arthritis diseases such as RA in humans [155].

## 6. Treatment Strategies in RA

### 6.1. First-Line Treatment

Current RA standard treatment includes the application of nonsteroidal anti-inflammatory drugs (NSAIDs), primarily used to control the pain and the inflammation, glucocorticoids (GCs), and disease-modifying anti-rheumatic drugs (DMARDs). Early-diagnosed RA patients may benefit from these therapies to alleviate inflammation and other disease symptoms, since these therapies target the inhibition of inflammatory mediators in order to treat the symptoms and prevent disease progression [159]. However, long-term administration of these drugs may cause adverse side effects such as gastrointestinal problems associated with vomiting, stomach ulcers, heartburn, or gastrointestinal bleeding [160,161]. More-target-specific therapeutics and novel strategies could help in minimizing these adverse effects and simultaneously increase treatment efficiency. Therefore, many different approaches have been developed or are currently under investigation, and are cited in the following section.

Table 1 provides an overview of the established RA treatment strategies as well as of novel approaches.

### 6.2. New Treatment Strategies

#### 6.2.1. Biological Agents

##### Targeting TNF by TNF Inhibitors (TNFi) in RA Therapy

Tumor necrosis factor is a central cytokine in the inflammatory cascade and plays a pivotal role in inflammatory diseases. Therefore, it is assumed to have also a significant impact on RA pathogenesis, where increased levels of TNF-α have been observed in the synovium as well as in the synovial fluid of RA patients [254] (Table 1). Consequently, the inhibition of TNF is an important target for symptom relief in RA treatment [162,163,255,256]. To date, five TNF-inhibitory drugs have been approved by the FDA and EMA and are used in the clinical setting for RA therapy [257]: Infliximab, a chimeric mouse–human monoclonal antibody (mAb); Etanercept, a soluble human dimeric TNF-receptor fusion protein; Adalimumab and Golimumab, fully human mAbs; and Certolizumab, a PEGylated, Fab’ only recombinant humanized antibody [162,256,258,259] (Table 1).

TNFi have led to significantly increased treatment efficacy and to improvement of the disease outcome in RA. Nevertheless, there are patients who have to stop TNFi treatment because of ineffectiveness or adverse reactions, such as an increased risk of infections, liver function abnormalities, hematological changes, and neurological disorders [162,179], and partially due to the synthesis of antidrug auto-antibodies accompanying TNFi administration [257,260].

##### Targeting IL by IL Inhibitors in RA Therapy

Similarly to TNF-α, interleukin also is a pro-inflammatory cytokine that contributes to the pathogenesis of RA by initiating and maintaining synovial inflammation and associated tissue destruction [190]. Therefore, different biological agents targeting ILs have been developed for medical use, including Anakinra and Canakinumab, both drugs that act as IL-1 inhibitors [190] (Table 1). In particular, IL-1 and IL-6 are considered as major mediators in RA inflammation and its pathologic effects. Moreover, studies found IL-1 to stimulate cartilage destruction and inhibit its matrix synthesis [186,261]. Anakinra, a recombinant form of a human interleukin-1 receptor antagonist, was the first biologic agent specifically developed for blocking IL-1 cell signaling in 1993 [186,190,262]. Canakinumab is a human monoclonal antibody that specifically binds to IL-1ß without any cross-reactions with other IL-1 family members [263]. Despite studies demonstrating evidence of inhibition of tissue destruction and improvement of clinical symptoms in RA patients by IL-inhibitor administration and its clinical effectiveness, studies also showed lower efficacy compared to TNFi [186,261,264]. Moreover, common side effects during treatment with IL inhibitors include, for example, urinary and respiratory tract infections, neutropenia, or erythema at the injection site [187,188].

In addition to the aforementioned IL-1 inhibitors, there are also drugs that inhibit other members of the interleukin family—for example, Tocilizumab, which is a recombinant humanized monoclonal antibody directed against soluble and membrane-bound interleukin 6 receptors, or Sarilumab, an mAb directed against IL-6 receptor [265,266]. Additionally, members of the interleukin 17 (IL-17) family, consisting of 6 structurally related cytokines, have received more attention (Secukinumab) as potential triggers in autoimmune diseases and playing a relevant role in inflammation, among others (i.e., IL-21, IL-12/23) [189] (Table 1).

##### Targeting Co-Stimulation by Co-Stimulation Blockers in RA Therapy

Another therapeutic strategy, besides those mentioned above, is the blockade of T-cell co-stimulation in order to modulate T-cell activation, since T-cells are abnormally active in RA [267]. The co-stimulation blocker Abatacept is one of the first selective co-stimulation modulators approved for RA therapy [268] (Table 1). Abatacept is a fusion protein assembled with the Fc region of human IgG1 and the extracellular domain of cytotoxic T-lymphocyte-associated antigen. With its properties of interfering with co-stimulation of T-cells by specifically binding to the co-stimulatory molecules CD80 and CD86 and thereby blocking interaction with CD28 on T-cells, Abatacept can selectively modulate and inhibit T-cell activation [268,269]. Although the described mode of action represents the main mechanism of Abatacept, it also influences other cell populations, including Tregs, monocytes, osteoclasts, and B-cells [194]. Abatacept represents a useful treatment alternative in RA and is mostly used for RA patients showing an inadequate response to previous therapy with at least one conventional DMARD including MTX or a TNFi [268,269]. Adverse effects commonly observed in patients undergoing Abatacept treatment are nasopharyngitis, headaches and nausea, hepatic disorders, and infections [195,196].

##### Targeting CXCL by CXCL Chemokine Inhibitors in RA Therapy

Chemokines are chemotactic cytokines that are involved in leukocyte recruitment in physiological as well as in pathological processes such as RA, where numerous chemokines are found at an increased level at inflammation sites such as the synovia [193,270]. In addition, they show many other functions, including cell proliferation and differentiation, cytokine production, angiogenic activities, and regulation of the adaptive immune response, as well as the development of immune memory, to name a few [192] (Table 1). With any imbalance in the chemokine network, their immunological control is at risk of failing and consequently of acting as a trigger for several disorders, including autoimmune diseases such as RA [192]. Various studies have demonstrated that the key role of chemokines for Th1 cells is to migrate into the synovium where chemokine ligands are profusely present. The inhibition of the chemokine receptors carried out in animal models showed a suppressing effect on inflammatory Th1 cells, which resulted in decreased synovitis [192,193]. In contrast, most human trials conducted with inhibitors targeting chemokine signaling pathways have not shown any improvements of clinical relevance [193].

##### Targeting B-Cells by Anti-B-Cell-Agents in RA Therapy

Besides other cell types, B-cells fundamentally contribute to the pathogenesis of RA through their antibody-dependent and also -independent functions, such as antigen presentation to T-cells in the synovia, secretion of important cytokines, and coordination of further inflammatory cells. Since all of these functions play a pivotal role in the disease of RA, the depletion of B-cells has become an effective mechanism of action in treatment strategies for RA [271] (Table 1). Therefore, many B-cell directed therapies have been designed, targeting B-cells in different ways. For example, Rituximab, Ofatumumab, Veltuzumab or Ocrelizumab, acting as CD20 Abs, and Epratuzumab, a CD22-targeting one, cause B-cell depletion through various mechanisms, including apoptosis, complement-dependent cytotoxicity, and mediation of antibody-dependent cellular cytotoxicity [198,199,200,201,202,272] (Table 1). Another common target in B-cells is the plasma membrane-embedded CD79, which is a target protein for pre-plasma cells and B-cells. Blocking CD79 leads to the inhibition of the BCR signaling pathway, resulting in the depletion of B-cells and germinal centers [273]. Another therapeutic approach in RA therapy is targeting CD40/CD154 (Table 1). Inhibition of the complex formation not only leads to attenuation of T-cell co-stimulation and B-cell stimulation, but also supports the conversion of CD4^+^ T-cells to Tregs [204,205,206]. Moreover, TLRs are assumed to contribute to the pathogenesis in RA, too, since they are highly expressed in the synovium of RA. In particular, the complex formation of TLR 7 and 9 has been demonstrated to induce B-cell activation; therefore, they represent another target in RA treatment strategies in order to inhibit binding between BCR and TLRs [205,208,209] (Table 1). Another approach for relieving RA symptoms is the inhibition of the transmembrane protein system of B-cell activating factor (BAFF) and a proliferation-inducing ligand (APRIL), which are both members of the TNF-family [210,274]. BAFF is produced by immune cells, including macrophages, neutrophils, dendritic cells, activated T-cells, stromal cells, and natural killer cells, and it is essential for B-cell maturation and survival [274]. In patients with autoimmune diseases, increased levels of BAFF and its homolog APRIL, are found, which might promote disease onset and prolongation [275]. Drugs such as Belimumab, a selective inhibitor of BAFF, or Atacicept, a dual inhibitor of BAFF and APRIL, are used to prevent B-cell differentiation and to rather promote B-cell depletion [210,211,268].

Furthermore, there are approaches to develop small-molecule inhibitors of inflammatory pathways in RA, such as, for example Fostamatinib (Table 1). Fostamatinib inhibits spleen tyrosine kinase (Syk), which is a vital non-receptor-type protein tyrosine kinase (PTK) that activates downstream MAPKs and the PI3K signaling pathway, leading to inflammatory effects. Syk plays an important role in the synthesis of FLS in patients suffering from RA; thus, its inhibition can contribute to reducing inflammatory symptoms [276]. Adverse effects of anti-B-cell agents are various, but commonly include a higher risk of infections, nausea, pruritus, and flushing [199,201]. Overall, strategies targeting B-cells show remarkable efficacy, as B-cells are involved in various mechanisms in autoimmune diseases such as RA. However, patient response to B-cell targeting treatment may vary due to disease and patient heterogeneity. For example, RA patients may have underlying heterogeneity in the synovium in terms of the degree of inflammatory cell infiltration, suggesting different B-cell subsets and their contribution to the disease. With this in mind, it is reasonable to speculate that a synovial-rich pathotype is more likely to respond to B-cell targeting than others. Therefore, B-cell targeting therapy may be more or less effective depending on the extent of B-cell depletion and the relative contribution of B-cells to disease [277].

#### 6.2.2. Synthetical Agents

##### Targeting Janus-Activated-Kinase (JAK) by JAK Inhibitors in RA Therapy

JAK signaling, an intracellular tyrosine kinase, has been identified as an important pathway among others in regulating the immune response. Through the JAK signaling pathway, many cytokines and other molecules such as interferons and growth factors can exert their functions, consequently contributing to the pathogenesis of various immune-associated disorders, including RA [278,279]. In contrast to healthy individuals, the JAK/STAT signaling pathway is dysregulated in RA patients, leading to continuous activation in RA synovial joints and consequently to increased levels of pro-inflammatory proteins in the inflamed synovial tissue [280].

During the last 10 years, numerous drugs targeting the JAK signaling pathway by its inhibition have been developed [278,279] (Table 1). In contrast to biological agents, JAK inhibitors do not target extracellular proteins, but aim to suppress intracellular ones [213,279] (Table 1). The JAK family consists of four members (JAK1, JAK2, JAK3, tyrosine kinase 2 (TYK2)) and the molecules, which are mediated by their pathway, exert their functions through type I and type II receptors. The receptors comprise different subunits that are associated with specific JAKs, a feature that was taken advantage of in newer JAK inhibitors, considering their specificity against selected JAKs [278]. The JAK-inhibitory drug Tofacitinib targets all JAKs (JAK1, JAK2, JAK3, and TYK2 (to a lower extent)) by acting as a competitive inhibitor of the ATP binding site of JAK, leading to the inhibition of JAK activation and its associated pathways. As a result, cytokine amounts as well as their production are reduced, and the immune response is modified [213]. In contrast to other JAK inhibitors such as Baricitinib (JAK1/JAK2), Upadacitinib, (JAK1) or Filgotinib (JAK1) only target some of the members of the JAK family, which can possibly be beneficial in terms of reducing adverse effects, which are comparable to those of biological drugs [278]. Overall, JAK inhibitors offer a promising perspective for the treatment of RA due to their broad spectrum of effector molecules that utilize the JAK/STAT pathway.

#### 6.2.3. Cell Therapy

##### Targeting Immunomodulation by MSC in RA Therapy

Since a number of RA patients are resistant or do not even show any response to established RA treatment strategies, therapy with MSCs provides an appealing opportunity based on the cells’ properties of self-renewal, regeneration of tissues, and the potential of modulating the immune response [281,282] (Table 1). The immunomodulatory effects of MSC comprise various mechanisms, including cell–cell contact, the transfer of extracellular vehicles containing messaging molecules, and the production of soluble factors including IL-1, Indoleamine 2,3-dioxygenase, transforming growth factor ß (TGF-ß), prostaglandin E2, and others [281,282,283]. Exerting their functions, MSCs are able to migrate to inflammatory sites, where they can act as suppressors of pro-inflammatory cytokine secretion as well as of the proliferation rate of B- and T-cells [281,282,283]. Furthermore, MSCs are able to modulate the differentiation of cells including monocytes, dendritic cells, macrophages, myeloid-suppressor cells, and neutrophils towards an immunosuppressive phenotype, making them a promising candidate for treating autoimmune diseases such as RA [281,282].

Underlining MSCs’ immunomodulating properties, studies showed that the expression of BAFF and APRIL cytokines was significantly decreased after MSC transplantation [284], while increased Tregs levels were found. In addition, diminishing amounts of anti-CCP Abs were observed in RA patients as well as a general clinical improvement [282].

##### Targeting Plasma Cell Depletion in RA Therapy

As stated before, B-cells are of great relevance in the initiation and progression of autoimmune disorders such as RA. Here, B-cells do not only play a role in inflammatory processes, but when differentiated into PCs, also synthesize pathogenic auto-antibodies, which often mediate autoimmune diseases [285,286].

In addition, current data showed that direct elimination of PCs could be beneficial in various autoimmune diseases, whereby it is important to differentiate between short- and long-lived plasma cells [285,287]. While short-lived plasmablasts are actually precursors of mature plasma cells, surviving only as long as B-cells are activated, long-lived plasma cells live in bone marrow and inflammation sites over months or even longer without the need for B- or T-cell contact. This background clarifies why B-cell targeting treatment does not affect mature plasma cells and emphasizes the importance of plasma cell-directed treatment approaches [218,288] (Table 1). To date, immunoablative therapy using ATG for stem cell transplantation and proteasome inhibitors are among the most promising therapies for efficiently depleting mature plasma cells [218,288]. The proteasome has a crucial role in the degradation of regulatory proteins and consequently displays a fundamental pillar in regulating cellular processes. Inhibiting the proteasome with drugs such as Bortezomib leads to the initiation of growth arrest and pro-apoptotic processes, contributing to PC apoptosis and depletion. Within the process of proteasome suppression, the release of pro-inflammatory NF-κB and its signaling pathways are also inhibited, outlining an important target for RA symptom relief [218,288].

#### 6.2.4. Combinational Therapy

Here, combinational therapy in RA describes the administration of biological agents with DMARDs, for example MTX (Table 1). There are clinical guidelines recommending a combinational treatment with both of them, when DMARDs are either not tolerated by RA patients, or if they do not show any response and DMARDs monotherapy is not effective. These guidelines were formulated after clinical trials showed evidence that concurrent use of biologics and DMARDs, for instance, TNFi and MTX, showed a significantly higher response than using one of them in monotherapy [178,227,228,287]. In addition, Tocilizumab as combinational therapy with MTX showed more advantages compared to tocilizumab monotherapy in patients who did not show a response to MTX monotherapy [228]. However, the administration of combinational treatment often depends on the individual response of patients and the severity of their prognosis [227,287].

#### 6.2.5. Targeting Epigenetic Factors in RA Therapy

Polyphenols

Epigenetic research has gained enormous importance and clinical relevance in the last decade. It is mainly concerned with the changes in gene expression and phenotypes of cells that are not related to changes in DNA sequence, which adds new quality and a whole new dynamic to the overview of gene expression. Epigenetic phenomena, such as DNA methylation, chromatin remodeling, post-translational histone modifications, and non-coding RNAs [289,290,291], are of fundamental importance in regulating gene expression in medicine, various diseases, and numerous processes such as cell proliferation and differentiation, development, cardiovascular diseases, diabetes, autoimmune diseases, and cancer [292,293,294,295].

In addition to several autoimmune diseases such as RA, epigenetic modifications have also been shown to increase inflammation in tissues through pro-inflammatory transcription factor nuclear factor kappa B (NF-κB) activation [296,297,298,299,300,301,302,303,304]. Furthermore, the epigenetic phenomena underlying the anti-inflammatory potential of diet and lifestyle conditions have resulted in the development of therapeutic actions to alleviate constant inflammation affecting the epigenome. In this context, biological components in the diet (polyphenols) have demonstrated anti-inflammatory effects through epigenetic mechanisms [305,306,307] (Table 1).

Curcumin (diferuloylmethane) is a natural polyphenolic compound found as a major component of turmeric (Curcuma Longa), which is used as a spice and has broad anti-inflammatory activity and proven benefits in the treatment of autoimmune diseases, including RA [240,308]. Based on studies, curcumin has vigorous anti-inflammatory, anti-oxidant, and anti-carcinogenic properties [309,310,311,312,313,314,315,316] and is a natural inhibitor of the pro-inflammatory transcription factor NF-κB, which mediates the regulation of inflammatory cytokines and proteins during OA and RA [299,309,317,318,319]. Curcumin has been reported to have consistently potent effects against RA as a multi-target agent. In fact, it has been reported that curcumin, a HAT-inhibitor, markedly diminishes histone H3 acetylation in the TNF-α-promoted IL-6 promoter, IL-6 mRNA, and IL-6 protein secretion in RA synovial fibroblasts [76]. In a pilot clinical trial, the overall efficacy and safety of curcumin in aggressive RA patients were evaluated, showing that curcumin significantly improved the American College of Rheumatology (ACR) Disease Activity Score (DAS) and was indeed well-established [240]. In addition, curcumin treatment has been shown to significantly reduce joint stiffness and swelling in patients with RA [320]. In fact, the specific anti-inflammatory effects of curcumin have been demonstrated in further clinical trials for the treatment of inflammatory orbital pseudotumors, too [321].

##### DNMT and HDAC Inhibitors

DNMT and HDAC inhibitors have been investigated for decades, but have been rediscovered recently for the treatment of inflammatory diseases, including RA (Table 1). Alterations and abnormalities in DNA methylation and histone acetylation have been shown to be present in RA, possibly contributing to its pathogenesis [81,322,323,324]. The enzymes DNMT and HDACs represent a class of important epigenetic regulators, and their inhibition offers the potential to reverse pathological conditions [81,325]. Inhibition of DNMT reactivates genes that were silenced by methylation and restores their normal function, while HDAC inhibitors increase acetylation, leading to higher DNA transcription activity, reducing cell proliferation, and initiating cell death [326,327]. Studies and clinical trials using the HDAC inhibitor Givinostat have already shown significant improvements in pathological conditions, including reduced pain and decreased levels of pro-inflammatory cytokines. Against this background, HDAC inhibitors represent another promising treatment strategy for RA. Common side effects of HDAC inhibitors include nausea, gastrointestinal and respiratory disturbances, and fatigue [81,324].

#### 6.2.6. Targeting Autophagy in RA Therapy

Various studies have demonstrated that autophagy and autophagy-related proteins are key players in immune regulation and play a pivotal role in autoimmune diseases, including RA. In addition, it has been observed that in mice, the deletion of these autophagy-related proteins leads to an improvement in RA disease and the prevention of joint destruction [328].

Since autophagy has a strong impact on immune functions by contributing to the removal of intracellular bacteria, the secretion of pro-inflammatory cytokines, antigen presentation, and lymphocyte spread, its modulation is considered as another target in RA therapy [147,328]. In order to modify autophagy, different therapeutics are used (Table 1). For instance, the administration of the autophagy-modulating drugs CQ and HCQ in clinical applications has been shown to be successful by inhibiting the lysosome, leading to suppression of T-cell activity and apoptosis resistance and reducing the characteristic increased levels of pro-inflammatory cytokines in RA [147,329]. Rapamycin represents another autophagy-targeting substance by activating autophagy and simultaneously inhibiting mTOR, which is activated in various inflammatory diseases [330].

#### 6.2.7. Treat-to-Target Strategy

**Figure 2:**
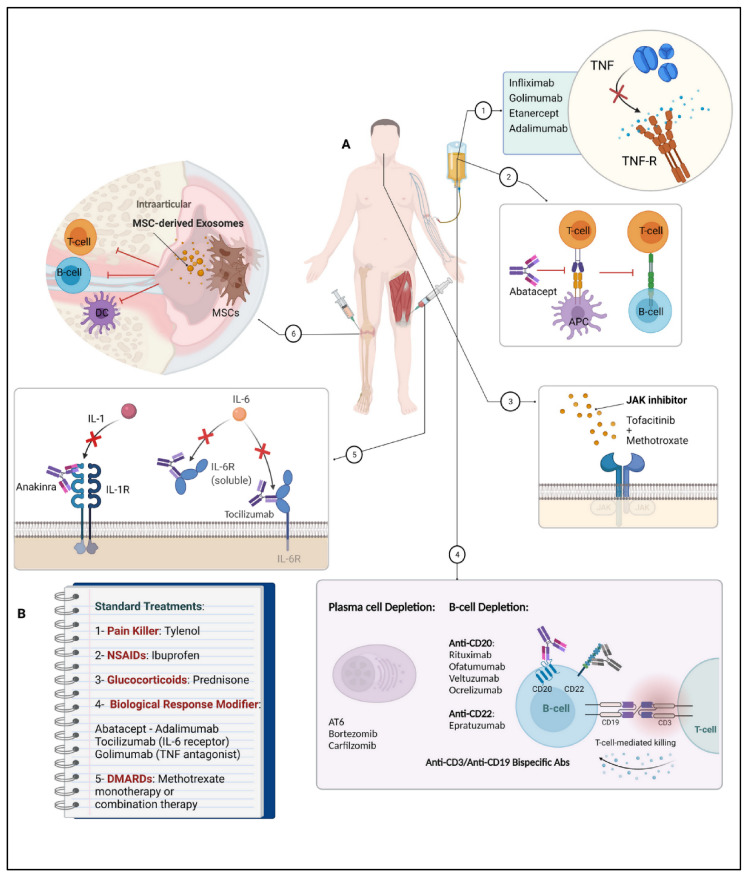
Treatment strategies for RA therapy. (**A**) New approaches in RA treatment. (1) TNF-inhibitors, (2) co-stimulation blockers (Abatacept), (3) Janus-Activated-Kinase (JAK) inhibitors (Tofacitinib + Baricitinib), (4) CD20 (Rituximab, Ofatumumab) and CD22 (Epratuzumab) targeting on B-cell surfaces and plasma cell targeting therapies (ATG), (5) IL-1 and IL-6 targeting mAbs (Anakinra and Tocilizumab), (6) intraarticular administration of mesenchymal stem cells. (**B**) Common standard treatments for RA patients (NSAIDs, GCs, biological response modifiers, and DMARDs).

First of all, the treat-to-target strategy theoretically comprises all treatment strategies in RA that are currently available for RA treatment with the aim of reaching a defined target by measuring disease activity and treatment progress regularly and adjusting treatment management if necessary [331,332] (Table 1). Treatment targets in RA involve low disease activity as well as remission. For monitoring disease activity, in order to make it quantifiable, defined measures are available as guidelines for treatment [333]. This strategy can help to gain control of the active disease as quickly as possible, and the number of treatment options have grown remarkably. Both the ACR and EULAR have described their treatment recommendations and guidelines, which show relevant similarities in the treatment of RA—for instance, the initial administration of MTX as monotherapy. However, the guidelines show differences too, not only in treatment strategies but also in the specification of disease stages and previous treatments. While the ACR considers early RA and DMARD-naive established RA separately, EULAR brings together both circumstances in a common guideline [163,178]. Nevertheless, disease activity should be reduced at least by half within 3 months, otherwise the current treatment approach should be modified [178]. Figure 2 shows an overview of various treatment strategies in RA.

## 7. Conclusions

The pathophysiology of RA is very complicated, and there is no patent remedy for the treatment and elimination of this systemic, multifactorial, inflammatory, and progressive autoimmune disease. The common drugs such as NSAIDs, GCs, DMARDs, biologics, and some others can primarily slow down the pain and inflammation, thereby preventing structural tissue defects and improving patients’ daily life. As the disease progresses, combination therapy is often required. However, when these drugs are taken chronically, numerous undesirable side effects can occur.

There is plenty of evidence in the literature that auto-antibodies, epigenetic changes, posttranslational modifications, and various other factors are involved in the progression of RA, but our understanding of the pathophysiology of RA remains tenuous. Although investigations have been ongoing for many decades, therapeutic interventions targeting various pathways have not yet been effectively used in the clinical setting. However, if pharmacogenetic and personalized medicine studies are accessible, side effects could be further minimized by treating patients with precision medicine methods. This would be possible by identifying valid biomarkers that can be used to find an adequate drug for every individual patient. On the other hand, novel techniques are necessary to identify different aspects of RA pathogenesis, such as cell (MSC therapy) and epigenetic therapy.

Currently, advances in understanding the molecular structure and mechanisms of phytopharmaceutical polyphenolic compounds such as curcumin and their abilities, particularly as potent anti-inflammatory and epigenetic modifying components, have mainly contributed to clinical trials in RA treatment, prevention, or concomitant administration with other drugs commonly used in RA therapy. In this paper, we reviewed the impact of these aspects on therapeutic strategies in RA, affecting the selection of therapeutic approaches and clinical endpoints.

## Figures and Tables

**Table 1:** Treatment strategies for RA therapy.

Standard Treatments			
**First-Line Treatments**	**Therapy**	**Mechanisms of Action**	**Refs.**
	**NSAIDs**	Inhibition of COX-1 and/or COX-2 enzymes, blocked formation of pro-inflammatory prostaglandins from the arachidonic acid metabolism, thereby interrupting the inflammatory cycle.	[160,161,162,163]
	*(i.e., Ibuprofen, Naproxen, Diclofenac, Etoricoxib, Celecoxib)*		
	Glucocorticoids (GCs)	Activation or suppression of protein synthesis, including cytokines, chemokines, inflammatory enzymes, and adhesion molecules by activating the cytosolic glucocorticoid receptor; thus, modification of immune responses and inflammatory mechanisms.	[164,165,166,167,168]
	*(i.e., Prednisone, Prednisolone, Budesonide)*		
	**DMARDs**	Impedes immune cell proliferation. Relieves symptoms such as joint pain and prevents joint damage.	[169]
	*(i.e., Methotrexate (MTX),*		
	*Hydroxychloroquine (HCQ),*	Interferes with deoxyribonucleotides metabolism, impairs the antigen presentation, and lysosomal membrane stabilization is increased.	[170]
	*Sulfasalazine,*	Anti-inflammatory, immunosuppressive effects, which are attributed to its breakdown products sulfapyridine and 5-aminosalicylic acid.	[171]
	*Leflunomide,*	Interferes with pyrimidine synthesis, which is needed for lymphocyte activation.	[172]
	*Azathioprine)*	Blocks purine metabolism.	[173]
**New/Future Treatment Strategies**			
**Biological Agents**	**Therapy**	**Mechanisms of Action**	**Refs.**
	**TNF inhibitors (TNFi)**	Selective inhibition of TNF-α, one of the major inflammatory cytokines, by specific binding to both its soluble subunit and its transmembrane precursor, leading to prevention of pro-inflammatory cell recruitment. Given the risk of developing Abs to the murine components of the molecule and the decreasing efficacy of monotherapy, RA therapy is only recommended in combination with MTX.	[162,174,175,176,177,178,179,180]
	*(i.e., Infliximab, Adalimumab, Golimumab, Certolizumab,*		
	*Etanercept)*	A biological TNF antagonist inhibiting the interaction of TNF-α and lymphotoxin (TNF-β) with cell-surface receptors, whereby TNF-mediated cellular responses and the activity of other pro-inflammatory cytokines are modulated.	[181,182,183,184]
	**IL-inhibitors**	Acting as IL-antagonists, thereby inhibiting cytokine binding to its receptors. Consequently IL-mediated signaling and its pro-inflammatory effects are suppressed.	[185,186,187,188,189,190,191]
	(*IL-1: i.e., Anakinra, IL-6: i.e., Tocilizumab, Sarilumab, IL-17: i.e., Secukinumab, IL-21 Abs*)		
	**CXCL chemokine inhibitors**	Inhibition of chemokines and chemokine receptors, which are found in inflamed synovia, to reduce the synovial migration of B-cells and the formation of ectopic germinal centers.	[192,193]
	*(i.e., CXCL-10, -12, -13)*		
	**Co-stimulation blockers**	Modulation of T-cell co-stimulation by binding to CD80 and CD86 receptors, thereby suppressing T-cell activation and B-cell stimulation.	[194,195,196,197]
	*(i.e., Abatacept)*		
	**Anti-B-cell-agents**	The monoclonal Abs cause depletion of B-cells through various mechanisms, i.e., apoptosis, complement-dependent cytotoxicity, and mediation of antibody-dependent cellular cytotoxicity.	[198,199,200,201,202,203]
	*(CD20: i.e., Rituximab, Ofatumumab, Veltuzumab, Ocrelizumab, CD22: i.e., Epratuzumab,*		
	*CD79 Abs,*	Targeting CD79 leads to inhibition of BCR signaling pathway, thereby depleting B-cells and ectopic germinal centers.	[204]
	*CD40/CD154 Abs,*	Blockade of complex formation leads to attenuation of CD154-mediated T-cell co-stimulation, inhibition of CD40-mediated B-cell stimulation, and supports CD4+ T-cells’ conversion to Tregs, mediating immunosuppression.	[205]
	*TLR7/9 Abs*	Inhibition of the binding between BCR and TLR (dominantly TLR7/9) leads to inactivation of B-cells.	[206]
	*BAFF: Belimumab*	Selective inhibition of BAFF (B-cell activating factor) prevents B-cell differentiation, thus leading to B-cell depletion.	[207,208,209]
	*BAFF/APRIL: Atacicept,*	Dual inhibitor of BAFF and APRIL (proliferation inducing ligand) by binding soluble BAFF and APRIL, thus interfering the interaction of these cytokines to their related receptors leading to B-cell depletion.	[210,211]
	*PTK/Syk: i.e., Fostamatinib)*	Inhibition of Syk, which is a vital non-receptor-type protein tyrosine kinase (PTK) activating down-stream MAPKs and the PI3K signaling pathway, leads to anti-inflammatory effects.	[212]
**Synthetical Agents**	**JAK-inhibitors**	Reducing the production of inflammatory cytokines by suppressing the JAK signaling pathway. Either as monotherapy or in combination with MTX for the treatment of active RA. Ensuring optimal use of JAK inhibitors in the treatment of RA in practice.	[213,214,215,216,217]
	*(i.e., Tofacitinib, Baricitinib, Upadacitinib, Filgotinib)*		
**Cell Therapy**	**Plasma cell therapy**	The polyclonal Abs with T-cell-depleting properties can modulate the immune response by affecting the function of various immune effectors including B-cells and Tregs.	[218]
	*(i.e., Anti-thymocyte-globuline (ATG),*		
	*Bortezomib, Carfilzomib)*	Proteasome inhibitor that suppresses activation of the pro-inflammatory transcription factor nuclear factor kappa B (NF-κB) by blocking degradation of the NF-κB inhibitor, thereby secretion of pro-inflammatory cytokines is reduced.	[219,220,221,222,223]
	**Mesenchymal stem cell (MSC) therapy**	Modulation of the immune response via cell-to-cell communication and MSC-secreted cytokines. For example, MSCs are able to directly inhibit T-cell function and differentiation or shifting them to functional Tregs.	[224,225,226]
**Combination Therapy**	**Combination of biologics and methotrexate**	There is much evidence that treatment with a combination of biologics and MTX is significantly more effective than treatment with biologics or MTX alone in some patients. Combination therapy is usually the treatment of choice when monotherapy with DMARDs is not effective.	[227,228,229]
	*(i.e., Tocilizumab and MTX Filgotinib and MTX)*		
**Epigenetic Therapy**	**DNA-methyltransferase (DNMT) and Histone-deacetylase (HDAC) inhibitors**	Modification of epigenetic marks such as hypomethylation and histone marks as the main treatment goal to restore abnormalities that contribute to the development of RA back to normal levels. Moreover, HDAC inhibitors’ effects are related to immune cell apoptosis.	[230,231,232]
	*(i.e., Givinostat, Vorinostat,* *Panobinostat, Romidepsin)*		
	**Polyphenols**	Therapeutic efficacy in the treatment of arthritis in CIA rats, reduction of inflammatory response by inhibition of NF-κB pathway resulted in significant relief of arthritic symptoms, by epigenetic regulation.	[233,234,235,236,237,238]
	*Curcumin*		
	*Curcumin and Prednisolone, or MTX or diclofenac, or Vitamin D3*	Combination therapy showed significantly higher therapeutic efficacy than simple treatment alone in patients with active RA and also in rats, by epigenetic regulation.	[239,240,241,242,243,244,245]
**Targeting Autophagy**	**Autophagy inhibitors**	Activation of autophagy by inhibiting mTOR.	[246,247]
	*(i.e., Rapamycin,*		
	*Chloroquine (CQ), Hydroxychloroquine (HCQ)*	Autophagy suppression by inhibiting the lysosome reduces the activity of T-cells and apoptosis resistance.	[248,249]
	*3-Methyladenine (3-MA)*	Inhibition of autophagy at an early stage of autophagosome development by blocking P13K signaling.	[250,251]
**Treat-to-Target**	**Monitoring of disease activity and adjusting therapeutic drug management**	Establishment of a treatment goal and measuring treatment progress and disease activity regularly, whereby the treatment strategy is adjusted until the goal is reached. The improvement of disease activity achieved in 3 months should be at least 50% by applying new therapeutic strategies, otherwise the treatment should be modified.	[252,253]

## Abbreviations

3-MA	3-Methyladenine
Abs	Antibodies
ACPA	Anti-Citrullinated Antibodies
ACR	American College of Rheumatology
ADA	Antidrug Auto-antibodies
AKT	Protein Kinase B
AMPA	Anti-Modified Protein Antibody
Anti-CarP	Antibodies targeting Carbamylated Proteins
Anti-RA33	Antibodies to the heterogeneous nuclear ribonucleoprotein A2/B1
APC	Antigen-Presenting Cell
APRIL	Proliferation-Inducing Ligand
ATG	Anti-Thymocyte Globulin
ATP	Adenosine Triphosphate
BAFF	B-cell Activating Factor
BCR	B-Cell Receptor
CCP	Cyclic Citrullinated Peptide
CD	Cluster of Differentiation
CD4^+^ CTL	CD4+ T-cells with Cytotoxic Potential
CIA	Collagen Induced Arthritis
CII	Collagen type II
CMV	Cytomegalovirus
COX	Cyclooxygenase
CQ	Chloroquine
CTLA4	Cytotoxic T-lymphocyte-associated Protein 4
CXCL	Chemokine Ligand
DNA	Deoxyribonucleic Acid
DAS	Disease Activity Score
DMARDs	Disease-modifying Anti-Rheumatic Drugs
DNMT	DNA-Methyltransferase
EA2	Endophilin A2
ELISA	Enzyme Linked Immunosorbent Assay
EMA	European Medicines Agency
EOMES	Eomesodermin gene
ER	Endoplasmic Reticulum
EULAR	European League Against Rheumatism
EV	Extracellular Vesicle
FDA	Food and Drug Administration
FLS	Fibroblast-Like Synoviocytes
FOXP3	Forkhead box P3
GC	Glucocorticoid
GWAS	Genome Wide Association Studies
HAT	Histone Acetyltransferase
HCQ	Hydroxy Chloroquine
HDAC	Histone Deacetylase
HLA	Human Leukocyte Antigen
HLA-DRB1	HLA class II Histocompatibility Antigen DRB1 beta chain
IFN	Interferon
IL	Interleukin
JAK	Janus-Activated Kinase
LC3-II	Microtubule-associated proteins 1A/1B light chain 3B
LTQ-ESI-MS	Linear ion-trap Electrospray Ionization Mass Spectrometry
mAb	Monoclonal Antibody
MHC II	Major histocompatibility Complex class II
MMP	Matrix-Metallo-Proteinase
mRNA	Messenger RNA
MSC	Mesenchymal Stem Cell
mTORC1	mTOR complex 1
MTX	Methotrexate
NADPH	Nicotinamide Adenine Dinucleotide Phosphate
NCF1	Neutrophil Cytosol Factor 1
ncRNA	Noncoding RNA
NF-κB	Nuclear Factor kappa B
NO	Nitric Oxide
NOS2	Nitric Oxide Synthase 2
NOX2	NADPH Oxidase 2
NSAID	Nonsteroidal Anti-Inflammatory Drug
OA	Osteoarthritis
PAD	Peptidyl Arginine Deiminase
PBMC	Peripheral Blood Mononuclear Cell
PC	Plasma Cell
PI3K	Phosphoinositide 3-Kinase
Prdm1	PR domain Zinc finger protein1
PTK	Protein-Tyrosine-Kinase
PTPN22	Protein tyrosine phosphatase, non-receptor type 22
QTL	Quantitative trait Loci
RA	Rheumatoid Arthritis
RA33	Heterogenous nuclear ribonucleoprotein A2/B1
RANKL	Receptor Activator of NF-κB Ligand
RF	Rheumatoid Factor
RNA	Ribonucleic Acid
ROS	Reactive Oxygen Species
SNP	Single Nucleotide Polymorphism
STAT3	Signal Transducer and Activator of Transcription 3
Syk	Spleen tyrosine kinase
TACI	Transmembrane Activator and CAML Interactor CD Antigen
TCR	T-cell Receptor
Tfh	Follicular T-helper Cell
TGF	Tumor Growth Factor
Th	T-helper Cell
TLR	Toll-Like Receptor
TNF	Tumor Necrosis Factor
TNFi	Tumor Necrosis Factor inhibitor
Treg	Regulatory T-Cell
TYK2	Tyrosine kinase 2
